# SHH pathway inhibition is protumourigenic in adamantinomatous craniopharyngioma

**DOI:** 10.1530/ERC-18-0538

**Published:** 2019-01-15

**Authors:** G Carreno, J K R Boult, J Apps, J M Gonzalez-Meljem, S Haston, R Guiho, C Stache, L S Danielson, A Koers, L M Smith, A Virasami, L Panousopoulos, M Buchfelder, T S Jacques, L Chesler, S P Robinson, J P Martinez-Barbera

**Affiliations:** 1 Developmental Biology and Cancer Programme, Birth Defects Research Centre, Great Ormond Street Institute of Child Health, University College London, London, UK; 2 Division of Radiotherapy and Imaging, The Institute of Cancer Research, London, UK; 3 Basic Research Department, Instituto Nacional de Geriatría, Mexico City, Mexico; 4 Division of Clinical Studies and Cancer Therapeutics Division, Paediatric Solid Tumour Biology and Therapeutics Team, The Institute of Cancer Research, London, UK; 5 Department of Histopathology, Great Ormond Street Hospital for Children, NHS Foundation Trust, London, UK; 6 Department of Neurosurgery, University Hospital Erlangen, Erlangen, Germany

**Keywords:** craniopharyngioma, pituitary, SHH, vismodegib, tumour

## Abstract

Pharmacological inhibition of the sonic hedgehog (SHH) pathway can be beneficial against certain cancers but detrimental in others. Adamantinomatous craniopharyngioma (ACP) is a relevant pituitary tumour, affecting children and adults, that is associated with high morbidity and increased mortality in long-term follow-up. We have previously demonstrated overactivation of the SHH pathway in both human and mouse ACP. Here, we show that this activation is ligand dependent and induced by the expression of SHH protein in a small proportion of tumour cells. We investigate the functional relevance of SHH signalling in ACP through MRI-guided preclinical studies using an ACP mouse model. Treatment with vismodegib, a clinically approved SHH pathway inhibitor, results in a significant reduction in median survival due to premature development of highly proliferative and vascularised undifferentiated tumours. Reinforcing the mouse data, SHH pathway inhibition in human ACP leads to a significant increase in tumour cell proliferation both *ex vivo*, in explant cultures, and *in vivo*, in a patient-derived xenograft model. Together, our results demonstrate a protumourigenic effect of vismodegib-mediated SHH pathway inhibition in ACP.

## Introduction

Adamantinomatous craniopharyngiomas (ACPs) are benign tumours of the sellar region that are associated with high morbidity and increased mortality in long-term follow-up. They constitute the most common non-neuroepithelial brain tumour in children (peak diagnosis at 5–14 years) and can also develop in adults (peak diagnosis 50–74 years). ACPs display clinically aggressive behaviour by invading vital surrounding structures such as the hypothalamus and optic chiasm, which complicates surgical resection leading to severe postoperative sequelae. Currently, there are no targeted molecular treatments for these patients (Karavitaki & Wass 2008, Muller *et al.* 2017) The majority of human ACPs carry somatic mutations in *CTNNB1*, which result in the expression of a degradation-resistant form of β-catenin and activation of the WNT/β-catenin pathway (Sekine *et al.* 2002, Kato *et al.* 2004, Buslei *et al.* 2005, Brastianos *et al.* 2014, Apps *et al.* 2018).

The expression of functionally equivalent mutant β-catenin in either pituitary embryonic precursors (*Hesx1* *^Cre/+^* *;Ctnnb1* *^lox(ex3)/+ ^*mice) or adult pituitary stem cells (*Sox2* *^CerERT2/+^* *;Ctnnb1* *^lox(ex3)/+ ^*mice) results in the formation of tumours resembling human ACP (Gaston-Massuet *et al.* 2011, Andoniadou *et al.* 2013). In both models, tumoural pituitaries show the presence of β-catenin-accumulating cells forming clusters, which share a common molecular signature with those found in humans (Gonzalez-Meljem *et al.* 2017). Our initial studies revealed the upregulation of the sonic hedgehog (SHH) pathway in both mouse and human ACP, a finding confirmed subsequently by independent research (Andoniadou *et al.* 2012, Gomes *et al.* 2015, Gump *et al.* 2015, Holsken *et al.* 2016). However, the biological function of the SHH pathway in ACP pathogenesis remains unknown to date.

The SHH pathway has been implicated in the pathogenesis of multiple cancers including medulloblastoma, basal cell carcinoma, breast, colon and pancreatic ductal adenocarcinoma (PDAC) (Rimkus *et al.* 2016). While SHH pathway inhibition has proven beneficial against basal cell carcinoma (Sekulic *et al.* 2012, 2017), it has proven detrimental in other cancers, such as PDAC, where enhanced tumour progression and aggressiveness was observed in both preclinical and clinical trials (Madden 2012, Catenacci *et al.* 2015). These discrepancies between favourable or unfavourable outcomes after SHH pathway inhibition prompted us to assess the functional relevance of the SHH pathway in human ACP. Our preclinical research demonstrates that inhibition of the SHH pathway using vismodegib is not beneficial and is therefore contraindicated in human patients.

## Materials and methods

### Mice

All experimental protocols were monitored and approved by The Institute of Cancer Research Animal Welfare and Ethical Review Body, in compliance with guidelines specified by the UK Home Office Animals (Scientific Procedures) Act 1986, the United Kingdom National Cancer Research Institute Guidelines for the Welfare of Animals in Cancer Research (Workman *et al.* 2010) and the ARRIVE guidelines (Karp *et al.* 2015). Animals were monitored daily and were killed at defined humane end points (i.e. prior to the mice showing signs of severe health deterioration or 20% weight loss). Patient-derived xenografts were generated as previously described (Stache *et al.* 2015). All mice were housed in compliance with the Home Office Code of Practice. Mice were kept on a 12-h light/darkness cycle and fed *ad libitum* with a complete pelleted mouse diet and with constant access to water.

*Hesx1* *^Cre/+ ^*mice have been previously described (Andoniadou *et al.* 2007). The line was maintained on a C57BL/6J background for over 50 generations. Heterozygotes were used for crosses with *Ctnnb1* *^lox(ex3)/+^* mice (Harada *et al.* 1999) to obtain *Hesx1* *^Cre/+^* and *Ctnnb1* *^lox(ex3)/+^* mice (Gaston-Massuet *et al.* 2011). For xenografts, we used NIH nu/nu were bred in our local Biological Services Unit.

### Drug administration

Four-week-old male *Hesx1* *^Cre/+^* *;Ctnnb1* *^lox(ex3)/+^* mice were administered vismodegib (Roche) or vehicle in 2% DMSO, 30% PEG 300, 5% Tween 80, ddH_2_O via oral gavage at a dose of 100 mg/kg of body weight twice a day (approximately 07:30 h and 16:00 h).

### Human samples

Experiments using human samples were covered by the ethical approval 14 LO 2265 or the ethical approval of specific tissue banks. Human ACP samples were kindly provided by the GOSH Histopathology Department.

### Ex vivo culture of ACP human tumours

Explant cultures were performed as previously described (Apps *et al.* 2018). Small pieces from three different human ACP tumours (approximately 1 mm^3^; four replicates per tumour) were placed on 0.2 μM Whatman filters (SLS) in 24-well plates containing 500 μL of media (DMEM-F12 (Gibco), 1% Pen/Strep (Sigma) and 1% FBS (PAA)) supplemented with either Vismodegib 100 μM (Selleckchem) or vehicle (DMSO) and medium was changed every 24 h. After 72 h, tumour pieces were passed through a Qiashredder column (Qiagen) and processed for total RNA extraction using the RNeasy Micro kit (Qiagen). Approximately 1 μg of total RNA was reverse-transcribed to cDNA using the Transcriptor First-Strand cDNA Synthesis Kit and random hexamers (Roche).

### Magnetic resonance imaging

Multi-slice T_2_-weighted images were acquired using a 7 T Bruker microimaging system with a 3 cm birdcage coil over a 2.5 cm field-of-view (RARE; T_R_ = 4500 ms, T_E_eff = 36 ms). MRI was performed on the final day of treatment and at least every 2 weeks thereafter, until mice presented with neurological symptoms or lost condition (Boult *et al.* 2018).

**Figure 1:**
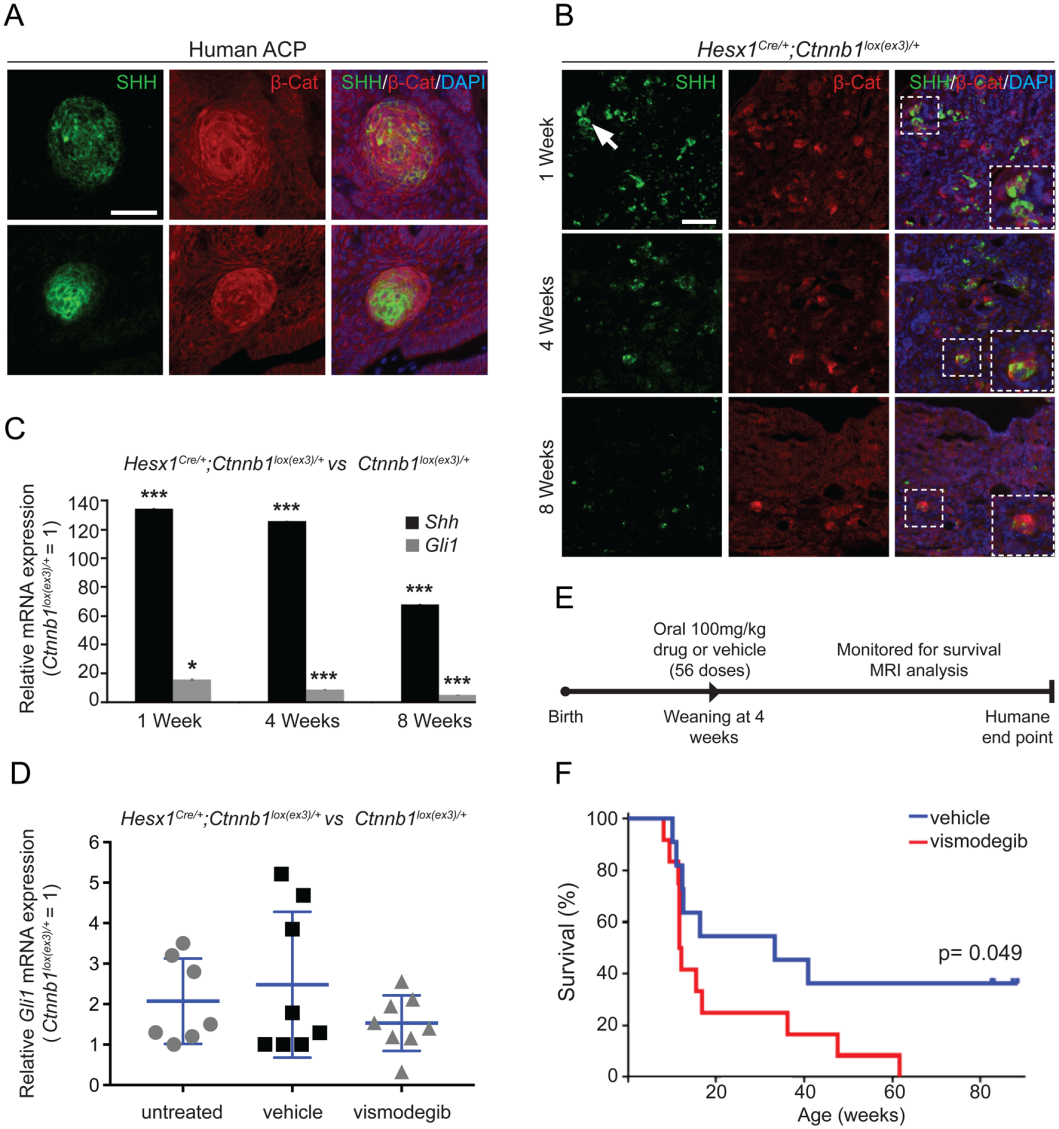
Inhibition of the SHH pathway in *Hesx1* *^Cre/+^* *;Ctnnb1* *^lox(ex3)/+ ^*mice, a murine model of human adamantinomatous craniopharyngioma (ACP), results in reduced median survival. (A) Double immunofluorescence on formalin-fixed paraffin-embedded (FFPE) histological sections of human ACP showing the expression of SHH in the β-catenin-accumulating cell clusters. Scale bar: 25 μm. (B) Double immunofluorescence on FFPE histological sections of *Hesx1* *^Cre/+^* *;Ctnnb1* *^lox(ex3)/+ ^*tumoural pituitaries revealing the expression of SHH in the β-catenin-accumulating cell clusters at specific time points of postnatal life. Note the overall reduction in SHH staining from 1 to 8 weeks of age. Scale bar: 50 μm. (C) qRT-PCR analysis showing the up-regulation of *Shh* and the target pathway gene *Gli1* in the tumoural pituitaries of the *Hesx1* *^Cre/+^* *;Ctnnb1* *^lox(ex3)/+ ^*ACP mouse model compared with *Ctnnb1* *^lox(ex3)/+ ^*controls at specific ages (*Shh*: 1 week, 134-fold; 4 weeks, 125-fold; 8 weeks, 68-fold; *Gli1*: 1 week, 15.3-fold, *P* = 0.018; 4 weeks, 8.7-fold, *P* < 0.0001; 8 weeks, 4.9-fold, *P* < 0.0001 (*n* = 3–5 tumoural pituitaries per time point, Student’s *t*-test). (D) qRT-PCR analysis of *Gli1* expression levels in *Hesx1* *^Cre/+^* *;Ctnnb1* *^loxex3/+^* tumoural and *Ctnnb1* *^lox(ex3)/+ ^*control pituitaries treated twice daily with vismodegib (100 mg/kg) for 1 week in comparison with vehicle-treated and untreated mice. There is an overall reduction in *Gli1* expression levels upon vismodegib treatment, which does not reach significance possibly due to the marked variability in *Gli1* expression levels in the untreated and vehicle treated controls (untreated: 2.1-fold, *n* = 7 mice; vehicle: 2.5-fold, *n* = 8 mice; vismodegib: 1.5-fold, *n* = 8 mice; *P* = 0.35, One-way ANOVA). (E) Schematic diagram of the preclinical trial. Age-matched *Hesx1 ^Cre/+^* *;Ctnnb1 ^loxex3/+^* male mice are dosed twice daily with 100 mg/kg vismodegib (11 mice) or vehicle (12 mice) for 28 days starting around 4–5 weeks of age (56 doses). At the end of treatment, mice are left untreated and monitored by MRI every 2 weeks and at the humane end point. (F) Kaplan–Meier analysis showing a reduction in median survival in the vismodegib-treated (11.9 weeks, *n* = 11, red line) relative to the vehicle-treated mice (33.3 weeks, *n* = 12, blue line; *P* = 0.049, Mantel–Cox (log-rank) test). Data represent the mean ± s.d.

### Histology and immunostaining on histological sections

Immunohistochemistry and immunofluorescence were carried out using the same antibodies, concentrations and retrieval conditions as previously described (Andoniadou *et al.* 2013, Gonzalez-Meljem *et al.* 2017, Apps *et al.* 2018). Immunostaining of SHH protein was also conducted as previously described (Carreno *et al.* 2017). Section immunostaining was performed as previously described (Andoniadou *et al.* 2012). Briefly, slides were de-waxed in HistoClear, re-hydrated from 100% EtOH to double distilled MilliQ water and underwent antigen retrieval in an antigen retrieval unit (BioCare Medical Decloaking Chamber NXGEN) for 2 min at 95°C. Slides were then washed in 1× PBT, which consists of 1× PBS and 0.1% Triton X-100. Histological slides were then blocked for 1 h at RT in blocking buffer and 10% heat inactivated sheep serum (HISS), blocking buffer contains 0.1% Triton X-100, 0.15% glycine, 2 mg/mL BSA in 1× PBS. SHH was visualised using the TSA Plus Fluorescein System (Perkin Elmer), following the manufacturer’s protocol. Primary and secondary antibodies were diluted into blocking buffer and 1% HISS. Sections were counter stained with 4′,6-diamidino-2-phenylindole (DAPI) for 5 min (1:10,000, Sigma) and mounted onto coverslips with VectaMount (Vector Laboratories). For immunohistochemical stainings, slides were first incubated with an Avidin-Biotinylated Peroxidase Complex (Vector). Chromogenic detection was then conducted by addition of 3,3′-diaminobenzidine (DAB, Vector) for 2–5 min and then counterstained with Mayer’s hematoxylin (Sigma).

### Quantitative analysis of immunofluorescent stainings

The proliferative and mitotic indexes were calculated as a percentage of the Ki67 positive or p-histone H3-positive cells out of the total of DAPI-stained nuclei, respectively. Cells were counted using ImageJ, a Gaussian filter was applied and the cell counter plugin was used. Endomucin-positive staining in pixels was analysed as a percentage of the total area using ImageJ.

**Figure 2:**
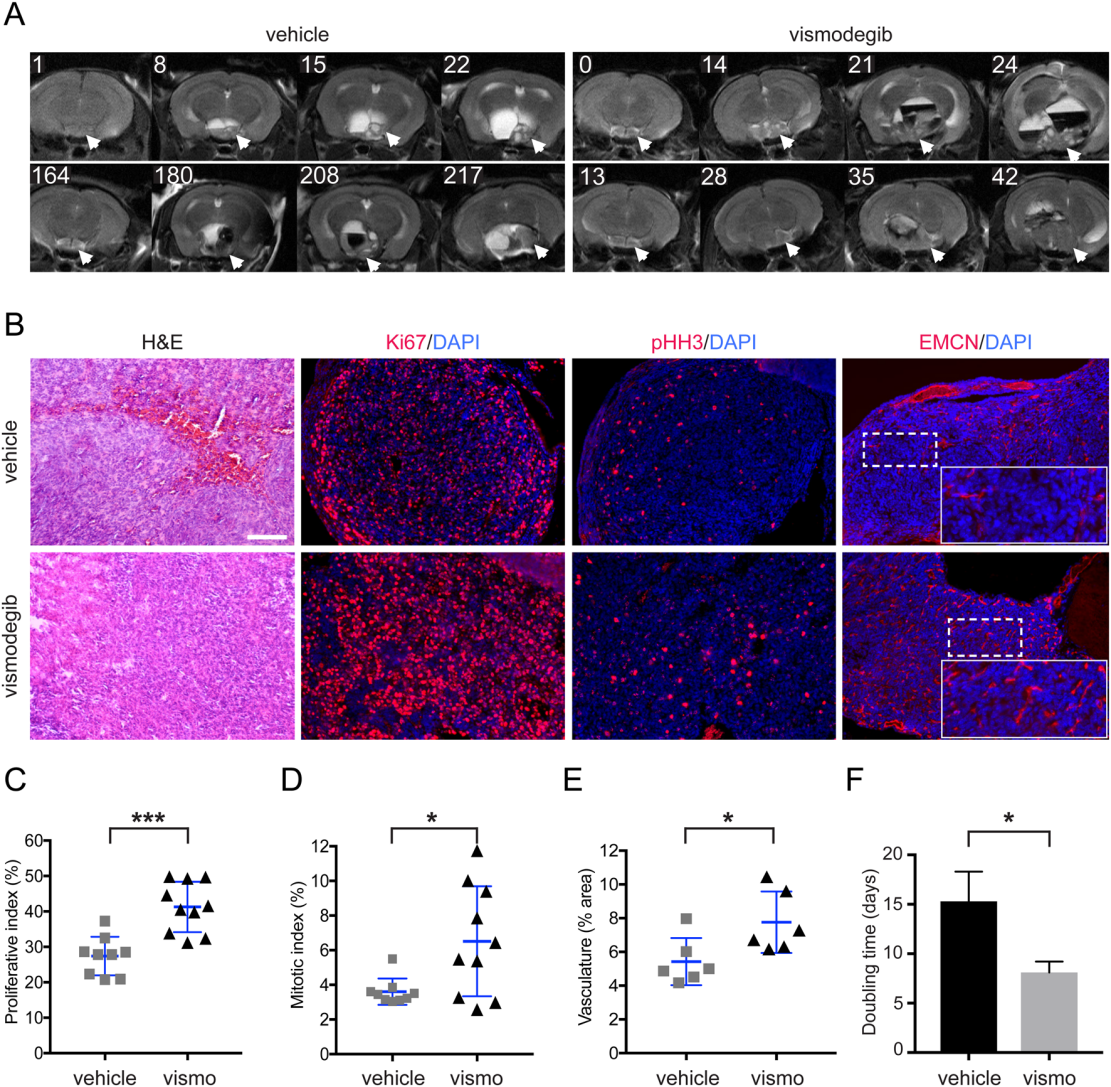
Highly proliferative and vascularised tumours are formed upon treatment with vismodegib in the *Hesx1* *^Cre/+^* *;Ctnnb1* *^lox(ex3)/+^* ACP mouse model. (A) Axial T2-weighted MRI scans of vismodegib and vehicle-treated *Hesx1* *^Cre/+^* *;Ctnnb1* *^lox(ex3)/+^* mice at the level of the brain (two examples of each are shown, labels indicate days after the end of treatment). Note the expansion of the pituitary to form a solid tumour component (arrows) and the development of hyperintense cysts and hypointense haemorrhagic regions in both vehicle and vismodegib-treated groups. (B) Hematoxylin–eosin (H&E) staining of FFPE histological sections of *Hesx1* *^Cre/+^* *;Ctnnb1* *^loxex3/+^* tumours showing similar histology between experimental groups. Immunofluorescence revealing the increased expression of Ki67 (proliferative marker), pHH3 (mitotic marker) and Endomucin (endothelial marker) in vismodegib-treated tumours compared with the vehicle-treated controls. Scale bar: 50 μm. (C) (vehicle: 27.4 ± 5.5%, *n* = 9 tumours; vismodegib: 41.3 ± 7.%, *n* = 10 tumours; *P* = 0.0002, Student’s *t*-test, 15,600 DAPI +ve nuclei counted). (D) The mitotic index (fraction of pHH3+ve cells out of total DAPI+ve cells) is elevated upon vismodegib treatment (vehicle: 3.6 ± 0.8%, *n* = 9 tumours; vismodegib: 6.5 ± 3%; *n* = 10 tumours; *P* = 0.016, Student’s *t*-test, 16,700 DAPI +ve nuclei counted). (E) Vasculature is increased in the vismodegib-treated group as assessed by immunofluorescence against Endomucin (total fluorescent area: vehicle: 5.4 ± 1.4%; vismodegib: 7.8 ± 2%; *n* = 6 tumours per group; *P* = 0.0319, Student’s *t*-test). (F) Doubling time of the solid component of the tumours, as calculated from contiguous MRI scans, is reduced by 47% upon vismodegib treatment (vehicle: 15.3 ± 3 days, *n* = 7 tumours; vismodegib: 8.1 ± 1 days, *n* = 6 tumours; *P* = 0.044, Student’s *t*-test). Data represent mean ± s.d.

**Figure 3:**
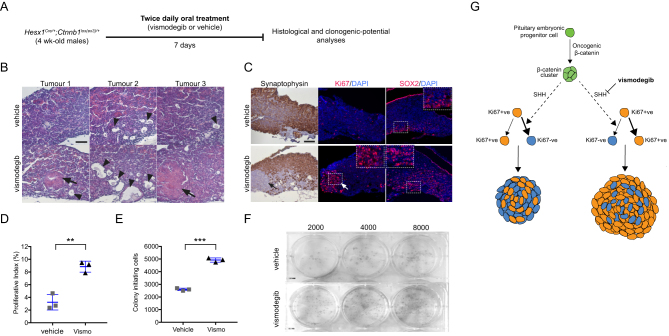
Vismodegib treatment results in higher numbers of clonogenic cells and premature formation of tumours. (A) Diagram of the experimental approach. Four-week old *Hesx1* *^Cre/+^* *;Ctnnb1* *^loxex3/+^* male mice are dose twice daily with either vismodegib (100 mg/kg) or vehicle for 1 week, after which pituitaries are dissected and analysed both histologically and in a clonogenic assay. (B) H&E staining on FFPE histological sections of vehicle and vismodegib -treated mice showing the presence of large tumoural lesions (arrows) and cysts (arrowheads) in vismodegib-treated animals. The control pituitaries show smaller cysts (arrowheads) and no tumour lesions are detectable (*n* = 3 pituitaries per group). This is in agreement with our previous observations of a latency period of around 17 weeks for tumour formation (Boult *et al*. 2018). Scale bar: 100 μm. (C) The premature tumour lesions are Synaptophysin−ve (black arrow) by immunohistochemistry, and highly proliferative, as assessed by immnufluorescence against Ki67 (white arrow). (D) Quantitative analysis demonstrates the higher Ki67 proliferative index upon vismodegib treatment (vehicle: 3.2 ± 1.2%; vismodegib: 8.8 ± 1.0%, *n* = 3 pituitaries per group; *P* = 0.0029, Student’s *t*-test, vehicle = 15,213, vismodegib = 13,300 DAPI +ve nuclei counted). Scale bar: 200 μm. (E) Quantification of the clonogenic potential of pituitaries from mice treated with either vehicle or vismodegib (*n* = 3 pituitaries per group). Note that vismodegib treatment results in drastic increase in clonogenic potential of nearly 90% relative to the vehicle controls (vehicle: 2597 ± 98 colonies; vismodegib: 4924 ± 167 colonies, *n* = 3 pituitaries per group; *P* = 0.0003, Student’s *t*-test). (F) Representative examples of plates seeded with 2000, 4000 and 8000 pituitary cells from vehicle and vismodegib-treated *Hesx1* *^Cre/+^* *;Ctnnb1* *^loxex3/+^* mice. Plates are stained with haematoxylin. (G) Schematic summary of main findings. The expression of oncogenic β-catenin in *Hesx1* *^Cre/+^* *;Ctnnb1* *^loxex3/+^* mice results in formation of β-catenin-accumulating cell clusters, which secrete SHH and activate the pathway in surrounding tumour cells inducing a more quiescent phenotype characterised by exit of a proliferative, Ki67+ve state. vismodegib-mediated inhibition of the SHH pathway leads to more proliferative and aggressive tumours. All graph bars represent mean ± s.d.

**Figure 4:**
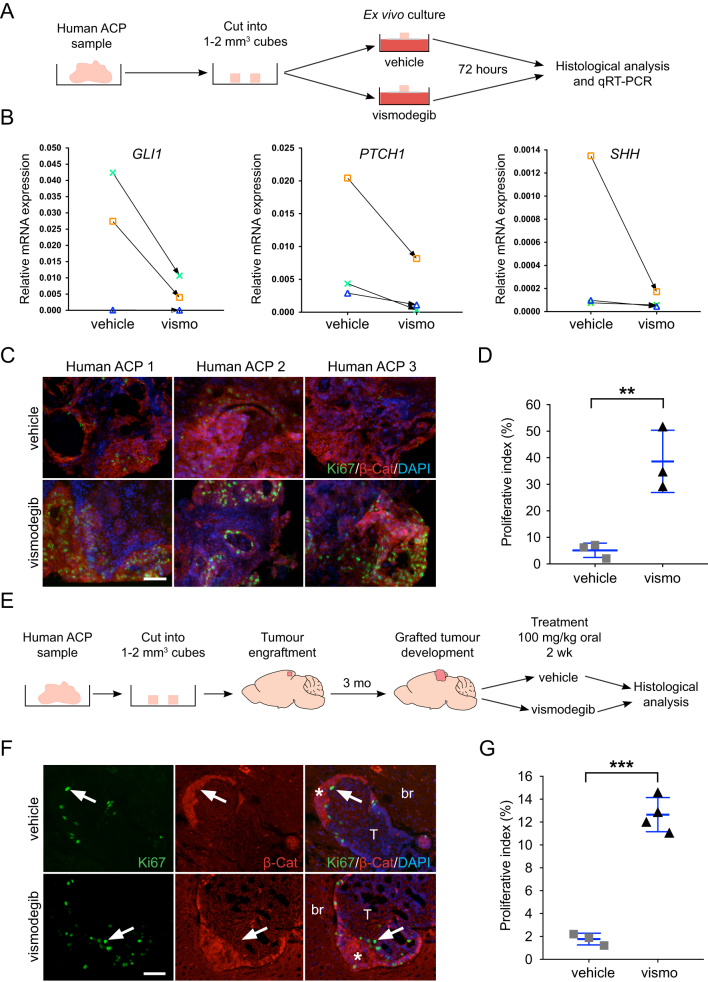
Inhibition of the SHH pathway leads to increased tumour cell proliferation in both explant cultures and xenograft models of human ACP. (A) Schematic diagram of the explant culture experiments. Three ACP tumour samples were cut into 1–2 mm^3^ cubes and cultured in the presence of vismodegib (100 μM) or vehicle (DMSO). Four biological replicates were performed for each analysis (i.e. four pieces of tumours per treatment). (B) qRT-PCR analysis showing the overall inhibition of the SHH pathway as assessed by a variable reduction in *GLI1*, *PTCH1* and *SHH* expression upon vismodegib treatment in three human ACP tumours. One sample did not show reduction of *GLI1*, but both PTCH1 and SHH expression were reduced. (C) Double immunofluorescence on FFPE histological sections showing increased expression of Ki67+ve in the human ACP explants cultured in the presence of vismodegib relative to the DMSO-treated controls. Scale bar: 25 μm. (D) Quantitative analysis revealing a higher Ki67+ve proliferative index in vismodegib compared with vehicle-treated explants (vehicle: 5.1 ± 3%; vismodegib: 38.7 ± 12%; *n* = 3 tumours per group; *P* = 0.0085, Student’s *t*-test, 10,037 DAPI +ve nuclei counted). (E) Schematic diagram of the human ACP xenograft experiments. Two human ACP tumours were cut into 1–2 mm^3^ and implanted into the cortex of 20 immunosuppressed mice (11 animals with tumour 1 and 9 with tumour 2). Three months later, mice were randomised into two groups of 10 mice each and dosed twice daily with 100 mg/kg vismodegib or vehicle for 21 days, after which brains were dissected and analysed histologically. (F) Double immunofluorescent staining revealing the presence of Ki67+ve cells (arrows) in the proximity of β-catenin-accumulating cell clusters (asterisks) in both vismodegib and vehicle-treated xenografted human ACP tumours. Scale bar: 25 μm. (G) Quantitative analysis showing an elevation of the Ki67 proliferative index in the xenografted tumours upon treatment with vismodegib (vehicle: 1.8 ± 0.5%, *n* = 3 tumour-bearing mice; vismodegib: 12.7 ± 2%, *n* = 4 tumour-bearing mice; *P* < 0.0001, Student’s *t*-test, 2332 DAPI +ve nuclei counted). All graph bars represent mean ± s.d.

### Quantitative reverse transcriptase PCR (qRT-PCR)

Quantitative reverse transcriptase polymerase chain reaction (qRT-PCR) amplification was performed on murine anterior pituitaries and human tumour samples as previously described (Gonzalez-Meljem *et al.* 2017, Apps *et al.* 2018). RNA was extracted from tissues using the RNeasy Micro or Mini Kit (Qiagen). RNA was quantified using 1.2 µL of extracted RNA on a NanoDrop 1000 Spectrophotometer. cDNA was retro-transcribed from 1 µg of extracted RNA using the iScript Reverse Transcription Supermix for RT-qPCR kit (BioRad). Thermocycling conditions were performed according to manufacturer’s instructions. qRT-PCR was performed using 64 iTaq Universal SYBR Green Supermix (BioRad), thermocycling conditions were performed according to manufacturer’s instructions. Analysis was performed using CFX Manager BioRad software. Three microliters of the amplified product was run by gel electrophoresis in order to visualise correct product size. Relative quantitation of the target gene was performed against glyceraldehyde 3-phospate dehydrogenase (*Gapdh*) as the housekeeping gene in murine and human samples. The average Ct values were calculated for the control and test sample genes. ΔCt was calculated by subtracting the Ct of the housekeeping gene from the target gene Ct values for both test and control samples. ΔΔCt was then calculated as the ratio of expression between the test and control samples. Finally, the fold change was calculated by 2−ΔΔCt for each sample. *SHH*, *GLI1* and *PTCH1* levels were measured using the Qiagen QuantiTect Primers (Cat. Qbib205625, Qbib60501, Qbib75824). Murine *Shh* and *Gli1* levels were measured with the Qiagen QuantiTect Primers (Cat. Qbib122479, Qbib173537).

### Murine pituitary adherent clonogenic cell culture

Clonogenic assays were performed on murine tumoural pituitaries as described (Gaston-Massuet *et al.* 2011, Carreno *et al.* 2017, Haston *et al.* 2017). Pituitaries were dissected using aseptic forceps and the posterior pituitary was removed. After mincing with forceps, the remaining tissue was placed into 200 µL of enzyme mix, which consisted of Hanks’ Balanced Salt Solution (HBSS, Gibco), 0.5% w/v Collagenase (Worthington), 50 µg/mL DNAase I (Worthington), 1% Fungizone and 0.1× trypsin (Sigma), for 4 h in a 37°C water bath. HBSS was added to make a final volume of 500 µL post incubation and the solution was triturated into a single-cell suspension. Once single-cell suspensions were achieved, 9.5 mL of HBSS was added and the cells spun down for 5 min at 200 ***g***. Cells were re-suspended in growth medium, which consisted of DMEM/F12, 5% FCS, 1% PenStrep, 20 ng/mL human recombinant bFGF (R&D Systems) and 50 ng/mL cholera toxin. Cells were plated at clonal density in a six-well plate at 2000, 4000 and 8000 cells per well. Fresh bFGF was added after 2 days, and medium was then changed on the third day and every 3 days after colony establishment. Colony counting was conducted after 7 days in culture. Colonies were washed with PBS and fixed for 20 min with 4% PFA, washed again in PBS and stained with Harris haematoxylin for 15 min at room temperature.

### Xenografts

Patient-derived xenograft (PDX) mice were generated as described (Stache *et al.* 2015). NIH nu/nu mice were anesthetised with 2% isoflurane and the top of the head was disinfected. An ~1 cm incision was made to the skin of the head to expose the skull. The incision was treated with 10% xylocaine. Thrity percent H_2_O_2_ was applied to the skull to remove the periosteal membranes. A 1 mm in diameter hole was bored into the skull, 1 mm left and 1 mm posterior of the Bregma. Human ACP tumour tissue was dissected into 1–2 mm^3^ pieces, and these were inserted into the cortex using forceps. The skin was then sutured to close the wound. The surgical area was disinfected again. Mice were placed on a heated mat and monitored until recovery.

### Statistics

Independent unpaired *t*-tests and one-way ANOVA were used to analyse the data from the qRT-PCR and proliferative assays using GraphPad Prism. Immunofluorescence was analysed using Image J. *P* < 0.05 was considered statistically significant. The ‘*n*’ value indicated throughout the text refers to number of biological replicas, that is, different mice, pituitaries or tumour samples. Technical replicas, that is, number of repeats for the same sample, were three for the qRT-PCR experiments. In general, we have not performed power calculations to estimate the sample size. In most of the quantitative experiments, we have used at least three biological replicas, often 6 or more. Only in the xenotransplantation experiments, have we used two human tumours. Many of these experiments use embryos or mouse and human tumours, which are samples not readily available. For the preclinical trial, we performed a power calculation, based on pilot experiments. A total of 12 mice per group were decided to compensate for sample attrition.

## Results

### SHH protein is expressed in mouse and human ACP leading to the paracrine activation of the SHH pathway

The expression of SHH at the mRNA level in the β-catenin-accumulating cell clusters and activation of the SHH pathway in human ACP have been thoroughly documented (Andoniadou *et al.* 2012, Gomes *et al.* 2015, Gump *et al.* 2015, Holsken *et al.* 2016). However, the SHH protein has not been identified in human or mouse ACP. Double immunostaining revealed the co-localisation of β-catenin and SHH protein in clusters of both human and mouse ACP (Fig. 1A and B, respectively). qRT-PCR revealed that *Shh* mRNA expression was increased in *Hesx1* *^Cre/+^* *;Ctnnb1* *^lox(ex3)/+^* mutant mice relative to age-matched *Ctnnb1* *^lox(ex3)/+ ^*controls at one (134 ± 1-fold), four (125 ± 1-fold) and eight (68-fold) weeks of age (*n* = 3–5 samples/group, *P* < 0.0001, Student’s *t*-test). The expression of the SHH pathway target gene *Gli1* was also upregulated at all stages in mutant pituitaries compared to controls (1 week: 15.3 ± 0.2-fold *P* = 0.018; 4 weeks: 8.7 ± 0.1-fold; 8 weeks: 4.9 ± 0.3-fold; *n* = 3–5 samples/group; *P* < 0.0001; Student’s *t*-test) (Fig. 1C). Note that a similar decreasing trend in *Gli1* and *Shh* mRNA expression was observed over time (Fig. 1C). Since mutations in the components of the SHH pathway have not been identified in humans (Brastianos *et al.* 2014, Apps *et al.* 2018) or mouse ACP (Gonzalez-Meljem *et al.* 2017), our data suggest that the SHH pathway is activated in ACP in a ligand-dependent manner.

### Inhibition of the SHH pathway in a genetically modified murine model of ACP results in reduced survival and increased tumourigenesis

To assess the role of the SHH pathway in the pathogenesis of ACP, we performed a MRI-guided preclinical trial using the smoothened (SMO) inhibitor vismodegib in the *Hesx1* *^Cre/+^* *;Ctnnb1* *^lox(ex3)/+ ^*ACP mouse model. Clinical trials have demonstrated that treatment with vismodegib results in long-term responses in patients with advanced basal cell carcinoma (Sekulic *et al.* 2012, 2017), and this inhibitor is currently being used in numerous ongoing clinical trials for other human cancers (Rimkus *et al.* 2016).

Pharmacokinetic studies in mice have shown that serum levels of vismodegib decrease after 12 h and suggested that a twice-a-day dosing regimen leads to more permanent pathway inhibition (Wong *et al.* 2011). An administration dose of 100 mg/kg of body weight was chosen as it is the highest non-toxic dose identified in the literature (Wong *et al.* 2011). A dosing regimen of twice a day for 7 days was chosen as it was hypothesised that any pharmacological effect on the SHH pathway would be evident after 7 days. This dosing regimen revealed an overall reduction in relative *Gli1* mRNA levels in the vismodegib-treated tumoural pituitaries compared to vehicle-treated control pituitaries, which did not reach significance (vehicle: 2.5-fold, vismodegib: 1.5-fold, *n* = 8; *P* = 0.35, ANOVA) (Fig. 1D). We noted a high degree of variability in *Gli1* expression levels in the vehicle-control group as well as untreated *Hesx1* *^Cre/+^* *;Ctnnb1* *^lox(ex3)/+ ^*mice (2.1-fold relative to *Ctnnb1* *^lox(ex3)/+ ^*controls, *n* = 7 mice), with some animals showing much higher *Gli1* levels than others, suggesting a similarly inherent heterogeneity in the ACP mouse model to that reported in human ACP (Gomes *et al.* 2015). *Gli1* expression mRNA levels were lower and more uniform with vismodegib treatment. We did not evaluate GLI1 expression at the protein level.

For the preclinical trial, *Hesx1* *^Cre/+^* *;Ctnnb1* *^lox(ex3)/+ ^*mice were dosed twice a day with 100 mg/kg of body weight of vismodegib or vehicle (11 and 12 mice, respectively) for a total of 56 doses over 28 days (the maximum allowed by UK animal welfare authorities) (Fig. 1E). Treated mice were monitored by MRI and killed at a defined humane endpoint (i.e. prior to the mice showing signs of severe health deterioration or 20% weight loss). Surprisingly, a significant decrease in survival was observed in the vismodegib-treated animals in comparison with vehicle-treated controls (11.9 weeks vs 33.3 weeks; *P* = 0.049, log-rank test) (Fig. 1F).

T_2_-weighted MRI throughout the trial up until each humane end point for both treated and vehicle groups demonstrated no significant differences in tumour phenotype and size of total and solid tumour volume (Fig. 2A). In other words, the vismodegib-treated mice did not develop larger tumours; at the humane end point, tumours were similar in size. MRI revealed tumour enlargement and heterogeneity in both groups prior to the development of hyperintense cysts, expansion of a solid portion of the tumour and presentation of hypointense haemorrhagic regions, consistent with our previous imaging studies (Boult *et al.* 2018). Furthermore histologically, tumours showed no gross morphological differences between the groups (Fig. 2B). However, the MRI data revealed that the doubling time of the solid component of the tumours was shorter in vismodegib-treated tumours compared with controls (vismodegib: 8.1 ± 1 days; *n* = 6; vehicle: 15.3 ± 3 days; *n* = 7; *P* = 0.044; Student’s *t*-test) (Fig. 2F). In agreement with these observations, the Ki67 proliferative index was increased in the vismodegib-treated group (41.3 ± 7%, *n* = 10) compared with controls (27.4 ± 5.5%, *n* = 9) (*P* = 0.0002, Student’s *t* test) (Fig. 2C). The mitotic index, measured by phospho-Histone H3 (pHH3) immunofluorescence, was also increased in the vismodegib-treated group (vismodegib: 6.5 ± 3%; *n* = 10; vehicle: 3.6 ± 0.8%; *n* = 9; *P* = 0.016; Student’s *t*-test) (Fig. 2D). Vismodegib treatment led to increased vasculogenesis, as assessed by immunofluorescence staining against the endothelial marker endomucin (fluorescent area: 7.8 ± 2% in vismodegib-treated group; 5.4 ± 1.4% in control group; *n* = 6; *P* = 0.0319; Student’s *t*-test) (Fig. 2E). Together, these results suggest that chemical SHH pathway inhibition using vismodegib in *Hesx1* *^Cre/+^* *;Ctnnb1* *^lox(ex3)/+ ^*mice results in the formation of aggressive tumours.

### Vismodegib-treated Hesx1^Cre/+^;Ctnnb1^lox(ex3)/+^ mouse pituitaries develop tumours prematurely and show increased numbers of clonogenic cells

We hypothesised that inhibiting the SHH pathway could lead to an increase in the number of cells with clonogenic tumour cells within, potentially tumour-initiating cells. Four-week-old *Hesx1* *^Cre/+^* *;Ctnnb1* *^lox(ex3)/+ ^*mice were treated with either vismodegib (100 mg/kg of body weight) or vehicle for 7 days, after which tumoural pituitaries were dissected and subjected to either histological analysis or assessment of the clonogenic potential as previously described (Gaston-Massuet *et al.* 2011, Andoniadou *et al.* 2013) (Fig. 3A). H&E staining identified tumour lesions in vismodegib-treated *Hesx1* *^Cre/+^* *;Ctnnb1* *^lox(ex3)/+ ^*pituitaries (3/3 pituitaries, Fig. 3B, arrows), which were not present in the vehicle-treated mice (0/3 pituitaries). These tumoural lesions were synaptophysin negative, suggesting loss of differentiation into hormone-producing cells (Fig. 3C), consistent with previous observations in mouse and human ACP. Furthermore, vismodegib-treated tumoural pituitaries showed a higher Ki67 proliferative index compared with controls (controls: 3 ± 1.2%; vismodegib: 8.8 ± 1.0%; *n* = 3; *P* = 0.0037; Student’s *t*-test) (Fig. 3D). We have previously shown the expansion of clonogenic SOX2-positive tumour cells in the *Hesx1* *^Cre/+^* *;Ctnnb1* *^lox(ex3)/+ ^*mice (Gaston-Massuet *et al.* 2011, Andoniadou *et al.* 2013). Immunofluorescent staining revealed an elevation in SOX2 expression in the vismodegib-treated *Hesx1* *^Cre/+^* *;Ctnnb1* *^lox(ex3)/+ ^*pituitaries compared with vehicle-treated controls. Moreover, dissociation of tumoural pituitaries into single-cell suspensions and culture in stem cell-promoting media showed a significant increase in the numbers of colonies in vismodegib-treated animals relative to controls (vehicle: 2597 ± 98; vismodegib: 4924 ± 167; *n* = 3; *P* = 0.0003; Student’s *t*-test) (Fig. 3E and F). Currently, there is no ACP primary cell culture that maintains the mutation sustaining cells throughout passages. Together, these results suggest that the inhibition of the SHH pathway leads to an increase in clonogenic tumour cells, with increased proliferative capacity, which promotes the development of premature tumour lesions (Fig. 3G).

### Inhibition of the SHH pathway in human ACP tumours leads to increased proliferation

Finally, we assessed the relevance of the murine studies in human ACP. Small pieces of human ACP tumours (1–2 mm^3^) were cultured *ex vivo* in the presence of vismodegib or vehicle, and after 3 days, the tissue was analysed (Fig. 4A) (Apps *et al.* 2018). Vismodegib treatment resulted in the overall downregulation of the SHH pathway, as assessed by qRT-PCR against *GLI1*, *PTCH1* and *SHH* (Fig. 4B), and the concomitant increase of the proliferative index (38.7 ± 12%) relative to the vehicle controls (5.1 ± 3%; *n* = 3; *P* = 0.0085; Student’s *t*-test) (Fig. 4C and D). To assess the effects of vismodegib *in vivo*, small fragments of human ACP were engrafted into the brains of 20 immunodeficient mice (Fig. 4E). After 3 months, mice were divided into two groups (10 mice/group) and administered 100 mg/kg of body weight of vismodegib or vehicle, twice a day for 3 weeks, after which brains were collected for histological analysis. Immunofluorescent staining revealed a significant increase in the Ki67 proliferative index in vismodegib-treated xenograft mice (12.7 ± 2%, *n* = 4) relative to the vehicle-treated controls (1.8 ± 0.5%, *n* = 3) (*P* < 0.0001, Student’s *t*-test) (Fig. 3F and G).

## Discussion

The main highlight of this research is the demonstration that the inhibition of the SHH pathway using vismodegib, a well-established SMO inhibitor used in the clinic (De Smaele *et al.* 2010), results in premature tumour formation and increased tumour cell proliferation in both genetically engineered and patient-derived xenograft mouse models. These data suggest that the use of vismodegib and potentially other SMO inhibitors is contraindicated in ACP patients.

The activation of the SHH pathway in human ACP was initially documented from research in the *Hesx1* *^Cre/+^* *;Ctnnb1* *^loxex3/+^* mouse model of ACP. The presence of mRNA expression was demonstrated in five human ACP tumours (Andoniadou *et al.* 2012). These results were extended further in a larger cohort of tumours by Gomes *et al*., who revealed the expression of GLI1, GLI3, SUFU and SMO in human ACP at the protein level (Gomes *et al.* 2015). More recently, the activation of the SHH pathway has been demonstrated in gene expression analyses of ACP (Holsken *et al.* 2016, Donson *et al.* 2017, Apps *et al.* 2018). Mutations in components of the SHH pathway (i.e. loss-of-function mutations in PTCH1 or gain-of-function mutations in SMO) have not been identified in independent sequencing efforts neither in human nor in mouse ACP, suggesting that the SHH pathway may be activated in a paracrine manner (Theunissen & de Sauvage 2009). Here, we extend these observations by demonstrating the presence of SHH protein within the beta-catenin-accumulating cell clusters, hence reinforcing the idea that the SHH pathway is activated in a ligand-dependent fashion. Of relevance, we have recently shown that these clusters contain senescent cells and act as signalling hubs within the tumours by activating the expression of numerous secreted factors, including SHH, FGF, EGF, BMP and TGF beta among others (Gonzalez-Meljem *et al.* 2017, Apps *et al.* 2018).

We show that treatment with vismodegib leads to accelerated tumourigenesis with tumours showing an elevated proliferation index, increased clonogenic potential *in vitro* and enhanced vascularity. In concordance with our preclinical data, research in mouse models of other human cancers, in which the activation of the SHH pathway is also ligand dependent, has revealed that the inhibition of this pathway (e.g. using vismodegib) is protumourigenic. For instance, the inhibition of the SHH pathway in pancreatic ductal adenocarcinoma (PDAC) murine models either chemically or genetically promotes tumour cell proliferation, undifferentiated phenotypes as well as increased vascularity, resulting in greater tumour burden and shorter survival (Lee *et al.* 2014, Rhim *et al.* 2014). Similarly, SHH pathway inhibition in colorectal cancer (CRC) murine models leads to higher numbers of cells with tumour-initiating potential and accelerated tumour formation (Madison *et al.* 2005, Gerling *et al.* 2016). Not unexpectedly, the inhibition of the hedgehog pathway in human PDAC and CRC was shown to yield rather disappointing results and patients either did not respond or, even worse, showed signs of faster cancer progression (Berlin *et al.* 2013, Catenacci *et al.* 2015, Ko *et al.* 2016). Moreover, a clinical trial using saridegib (an SMO inhibitor also known as IPI-926) in patients with PDAC was stopped due to increased tumourigenesis in the drug-treated group relative to the controls (Madden 2012, Rimkus *et al.* 2016) (http://investors.infi.com/static-files/a2cbb418-8048-4f1f-94cc-dbbff91245be).

A limitation of the research presented here is the absence of corroborating genetic data to further elucidate the possible mechanisms by which SHH pathway inhibition leads to increased tumourigenesis in ACP. Unfortunately, *Hesx1* *^Cre/+^* *;Shh* *^flox/^* *^−^* mice die at birth, which prevents such genetic study (i.e. the *Hesx1* *^Cre/+^* *;Ctnnb1* *^loxex3/+^*;*Shh* *^flox/^* *^−^* mice cannot be generated) (Carreno *et al.* 2017). Although ACP cellular models have been used, these have not been molecularly characterised and in our hands, the *CTNNB1* mutations that characterise the ACP tumours are lost in the culture cells. Nonetheless, our cellular and molecular characterisation suggest that common mechanisms underlie the protumourigenic effects of SHH pathway inhibition in ACP, PDAC and CRC. We propose that in addition to the angiogenic effects, such inhibition prevents the exit of the cell cycle, resulting in higher numbers of proliferative tumour cells and accelerated tumourigenesis (Fig. 3G). In summary, using a clinically approved and widely employed SMO inhibitor in both the *Hesx1* *^Cre/+^* *;Ctnnb1* *^loxex3/+^* mice and a human xenograft model, our research demonstrates that the inhibition of the SHH pathway has a tumour-promoting effect. We conclude that the use of vismodegib, and potentially other SMO inhibitors may be contraindicated in ACP patients.

## Declaration of interest

The authors declare that there is no conflict of interest that could be perceived as prejudicing the impartiality of the research reported.

## Funding

Funding for this research was provided by Cancer Research UK, Children’s Cancer and Leukaemia Group, Children with Cancer UK (15/190), MRC (MR/M125/1), Brain Tumour Charity (SIGNAL and EVEREST), Great Ormond Street Hospital Children’s Charity, Morgan Adams Foundation and National Institute of Health Research Biomedical Research Centre at Great Ormond Street Hospital for Children NHS Foundation Trust and University College London. The authors thank CR-UK support to the Cancer Imaging Centre at The Institute of Cancer Research and The Royal Marsden Hospital in association with the MRC and Department of Health (England) (C1060/A16464). S H is supported by a Wellcome Trust PhD Fellowship. J R A is supported by a Cancer Research UK Clinical Research Training Fellowship. J P M-B is a Great Ormond Street Hospital for Children’s Charity Principal Investigator.
